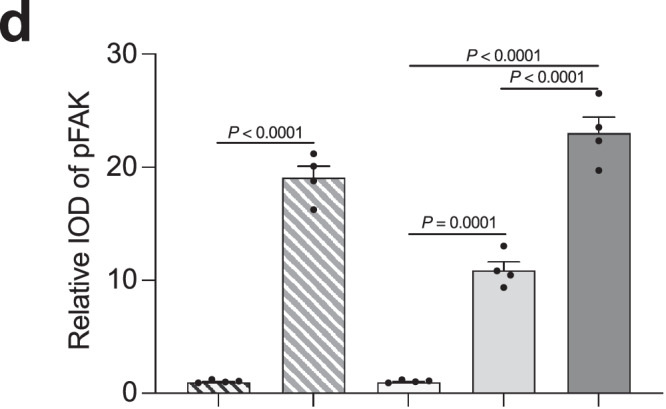# Author Correction: Hydrogel dressing integrating FAK inhibition and ROS scavenging for mechano-chemical treatment of atopic dermatitis

**DOI:** 10.1038/s41467-026-74416-y

**Published:** 2026-07-07

**Authors:** Yuanbo Jia, Jiahui Hu, Keli An, Qiang Zhao, Yang Dang, Hao Liu, Zhao Wei, Songmei Geng, Feng Xu

**Affiliations:** 1 The Key Laboratory of Biomedical Information Engineering of Ministry of Education, Xi’an Jiaotong University School of Life Science and Technology, 710049 Xi’an, China; 2 Bioinspired Engineering and Biomechanics Center (BEBC), Xi’an Jiaotong University, 710049 Xi’an, China; 3 Department of Dermatology, The Second Affiliated Hospital, Xi’an Jiaotong University, 710004 Xi’an, Shaanxi P. R. China

Correction to: *Nature Communications* 10.1038/s41467-023-38209-x, published online 29 April 2023

In the version of the article initially published, in Fig. 2d and the corresponding Source data, the mouse pFAK relative IOD quantification data were incorrect. The human pFAK quantification data shown in Fig. 2d were not affected. Fig. 2d has now been corrected in the HTML and PDF versions of the article, as seen in Fig. 1, below. Additionally, the Source data for Supplementary Fig. 2g was inadvertently duplicated from Supplementary Fig. 2e. The bar chart in Supplementary Fig. 2g was generated from the correct dataset and is not affected. The Source data for Fig. 2d and Supplementary Fig. 2g have been corrected and the updated Source data file is now available online.

**Fig. 1 Original and corrected Fig. 2d**

**Original Fig. 2d** 
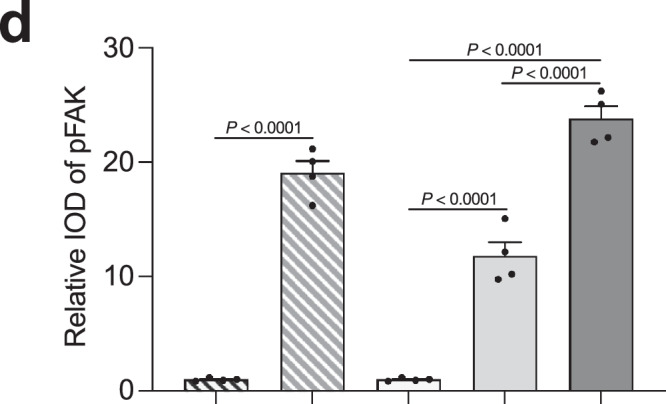


**Corrected Fig. 2d**