# Supplementary material for: Hydrogel dressing integrating FAK inhibition and ROS scavenging for mechano-chemical treatment of atopic dermatitis
Source: Nat Commun. 2023 Apr 29;14:2478. doi: 10.1038/s41467-023-38209-x (PMC10148840; doi:10.1038/s41467-023-38209-x)
Supplement: Supplementary file 1 — Supplementary Information [file 41467_2023_38209_MOESM1_ESM.pdf]

Hydrogel Dressing Integrating FAK Inhibition and ROS Scavenging for Mechano-chemical Treatment of Atopic Dermatitis

Yuanbo Jia[1,2#], Jiahui Hu[2,3#], Keli An[1,2], Qiang Zhao[2,3], Yang Dang[2,3] Hao Liu[1,2], Zhao Wei[1,2], Songmei Geng[3*], Feng Xu[1,2*]

[1] *The Key Laboratory of Biomedical Information Engineering of Ministry of Education, Xi'an Jiaotong University School of Life Science and Technology, Xi'an, China 710049*

[2] *Bioinspired Engineering and Biomechanics Center (BEBC), Xi'an Jiaotong University, Xi'an, China 710049*

[3] *Department of Dermatology, The Second Affiliated Hospital, Xi'an Jiaotong University, Xi'an, Shaanxi 710004, P. R. China*

[#] *The authors contributed equally.*

[*] *Corresponding authors: gsm312@yahoo.com; fengxu@mail.xjtu.edu.cn*

Supplementary Figures

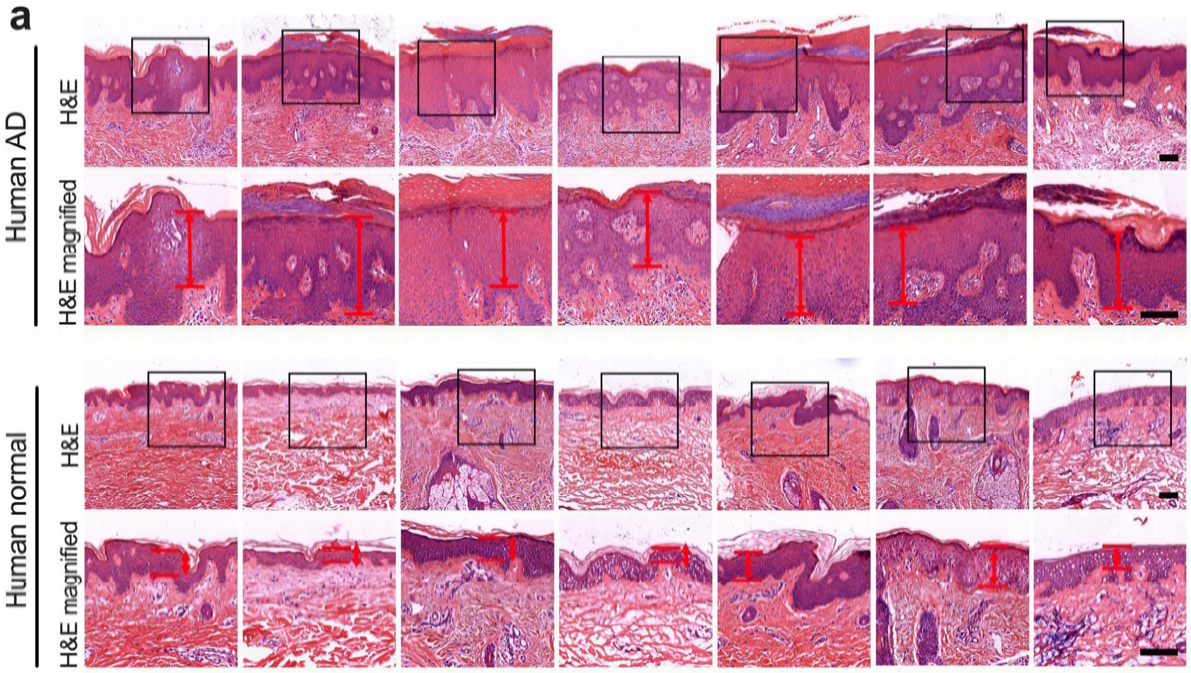

Supplementary Figure 1 | H&E staining of human skins.

a, H&E staining of all human skin sections. n = 8 human for each group. The black boxed area is enlarged below. The space between red lines denotes the epidermal thickness. Scale bar, 100 µm.

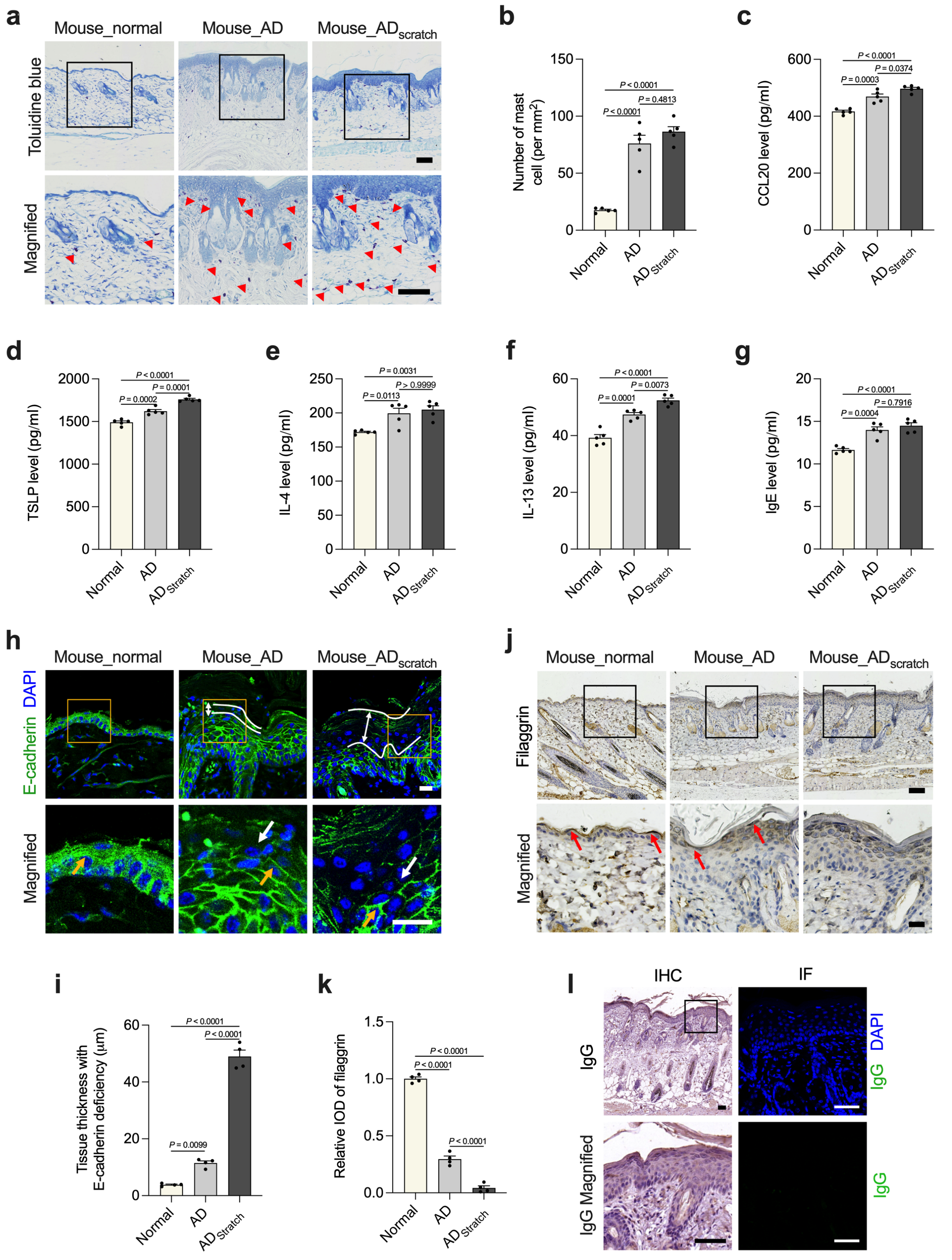

Supplementary Figure 2 | Evaluation of inflammation and epidermal barrier damage after scratching AD skins.

a, Representative toluidine blue staining of skin section. The red triangle denotes dermal mast cells. Scale bar, 100 μm. n = 5 mice. **b**, Measurement of the density of mast cells for each group

after treatments. n = 5 mice. **c-g,** CCL-20, TSLP, IgE, IL-4 and IL-13 levels in mouse tissues of each group, n= 5 mice. **h,** Immunofluorescent (IF) staining of E-cadherin (green) and DAPI (blue) in each group. Scale bar, 20 μm. The white lines denote the deficient area of E-cadherin in the epidermis. In the magnified images, the yellow arrows denote intact intercellular junctions represented by intact E-cadherin, while the white arrows indicate deficient E-cadherin. **i**, Quantification of tissue thickness with E-cadherin deficiency, n = 4 mice. **j**, Immunohistochemistry (IHC) staining of filaggrin in each group. Scale bar, 100 μm for the images above and 25 μm for the magnified images. The red arrows indicate the area of high DAB staining (high filaggrin expression). **k**, Relative IOD of filaggrin quantified from images of **h**, n = 4 mice. **l**, Representative images of IHC and IF staining of IgG. Scale bar, 50 μm. n = 3 tests with similar results. All data are shown as mean ± s.e.m., and compared by one-way ANOVA followed by Bonferroni's post hoc test, respectively.

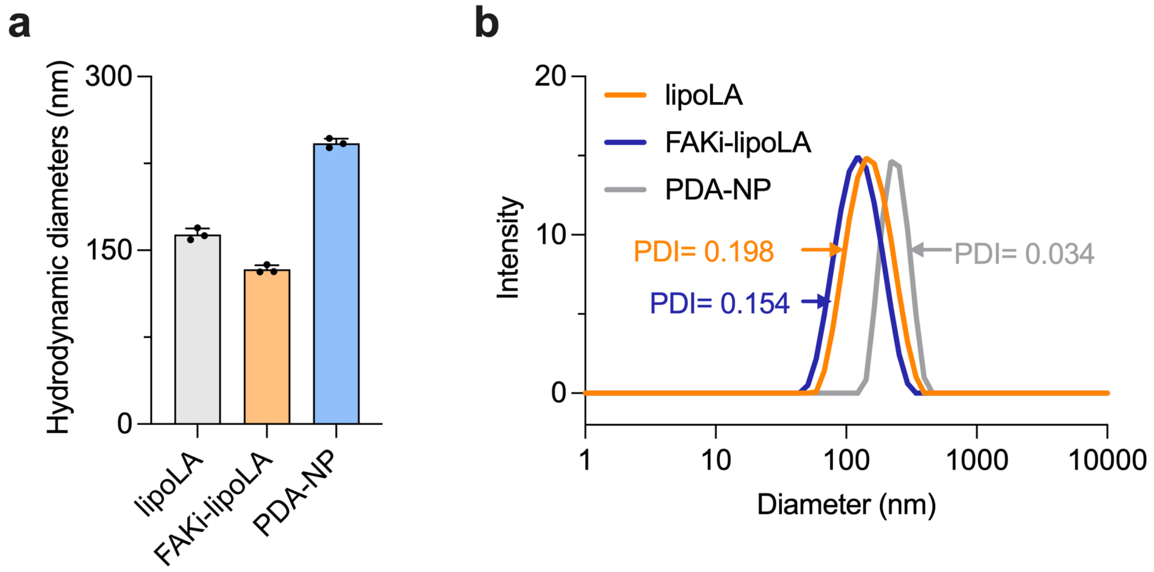

Supplementary Figure 3 | Characterization of PDA NPs and FAKi-lipoLA.

a, hydrodynamic diameters of different particles. n = 3 samples. Data are shown as mean ± s.d..

b, particle dispersity index (PDI) of different particles.

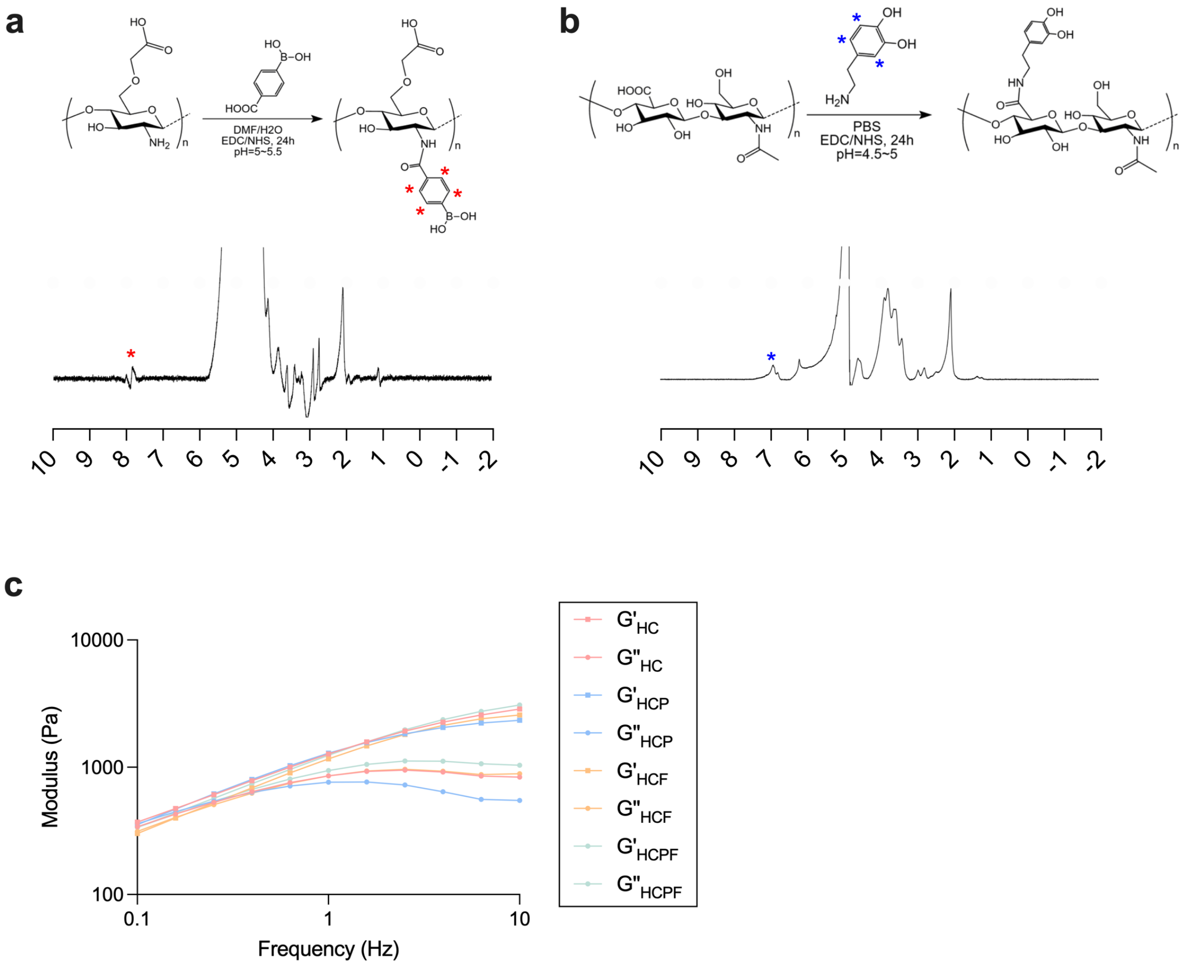

Supplementary Figure 4 | Synthesis and ^{1}H NMR spectra of HADA and CMCS-PBA.

a, Synthesis and ^{1}H NMR spectra of CMCS-PBA. **b**, Synthesis and ^{1}H NMR spectra of HA-DA. **c,** Representative curve of frequency sweep test of different hydrogels.

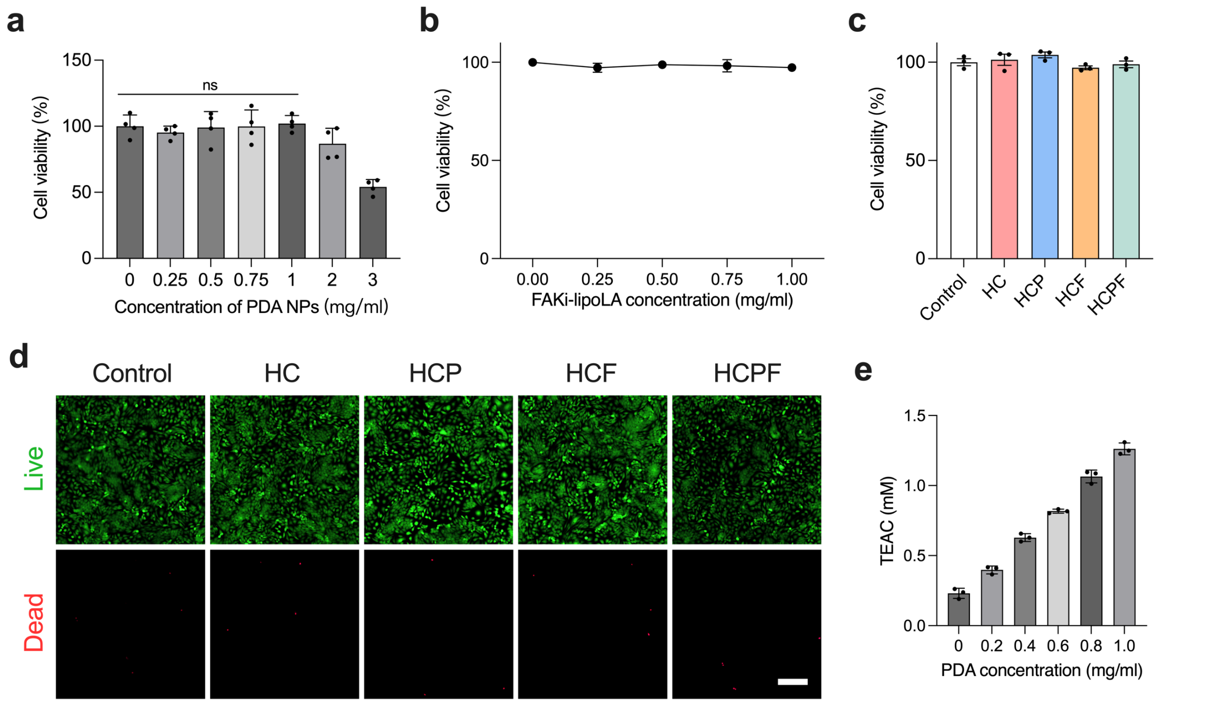

Supplementary Figure 5 | Cell compatibility of PDA NPs, FAKi-lipoLA and HCPF hydrogels.

a-c, Cell viability of PDA NPs, FAKi-lipoLA and different hydrogels measured by MTT assay. n=3 for each group. All data are shown as mean ± s.e.m. and compared by one-way ANOVA followed by Bonferroni's post hoc test, respectively. **d**, Live/dead staining of HaCaT cells with Calcein (AM, green) and propidium iodide (PI, red). n = 3 tests with similar results. Scale bar, 100 μm. **e**, Total antioxidant efficiency of HCP hydrogels with PDA NPs of different concentrations, n = 3 samples, data are shown as mean ± s.d..

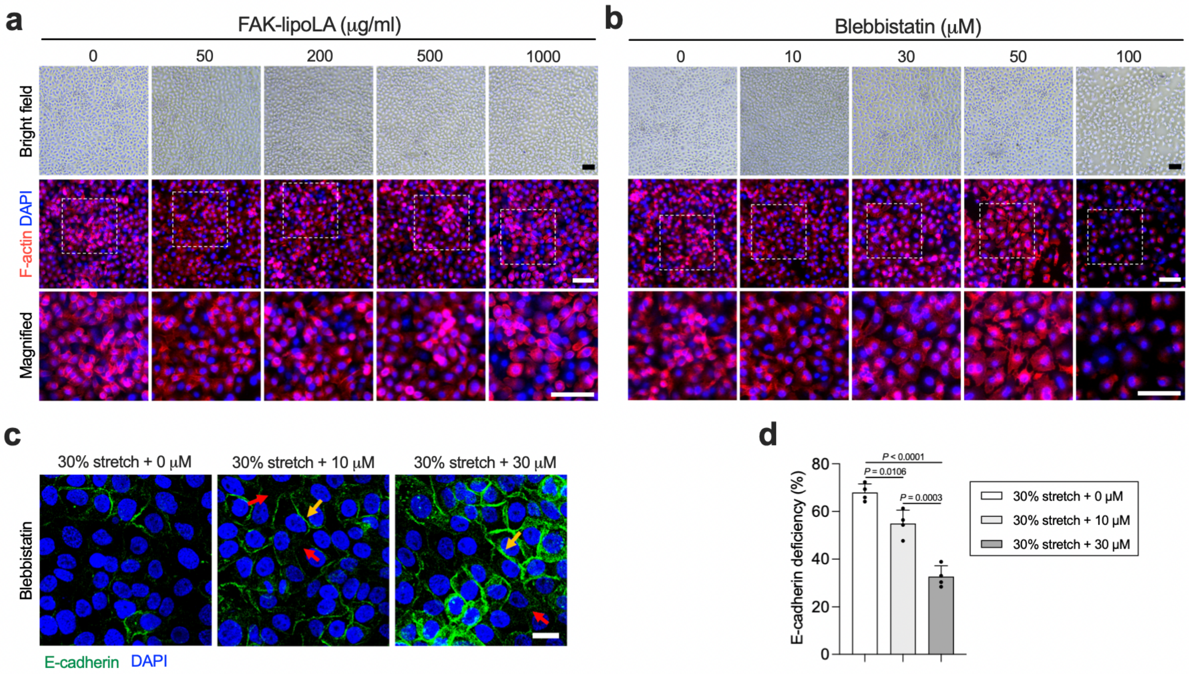

Supplementary Figure 6 | Effect of blebbistatin on cell morphology and E-cadherin under stretch.

a-b, HaCaT cell morphology after treatment with different concentration of FAK-lipoLA and blebbistatin. Cells are stained with F-actin (red) and DAPI (blue). n = 3 tests with similar results. Scale bar, 100 μm. **c**, Fluorescent staining of HaCaT cells after stretching with E-cadherin (green), F-actin (red) and DAPI (blue). Scale bar, 50 μm. **d**, E-cadherin deficiency of HaCaT cells calculated from staining images in **c**. n = 4. Data are shown as mean ± s.e.m. and compared by one-way ANOVA followed by Bonferroni's post hoc test.

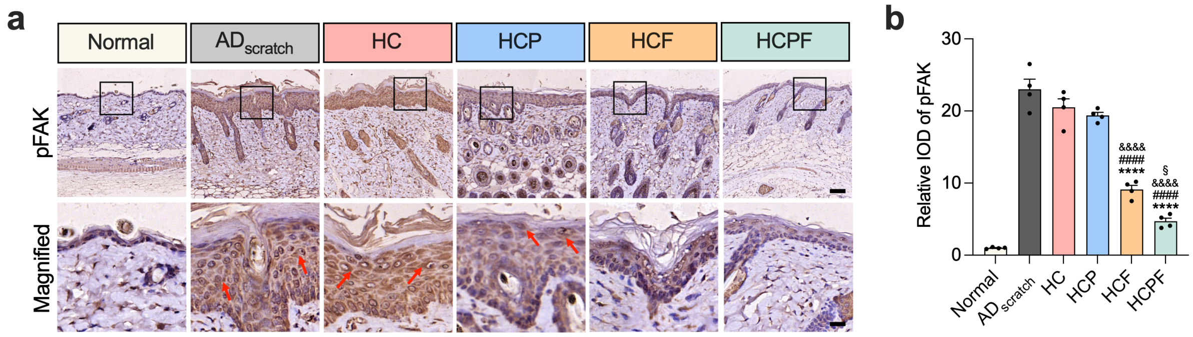

Supplementary Figure 7 | Assessment of FAK phosphorylation levels in tissues.

a, Immunohistochemistry staining of pFAK in each group. Scale bar, 100 µm for the images above and 25 µm for the magnified images. The red arrows indicate the area of high DAB staining (high pFAK expression). n = 4 mice with similar results. **b**, Relative IOD of pFAK quantified from images of **a**, n = 4 mice. All data are shown as mean ± s.e.m.. *, #, & and § indicates data compared with HC, HCP, and HCF, respectively. * and **** indicate $P < 0.05$ and $P < 0.0001$ compared by one-way ANOVA followed by Bonferroni's post hoc test, respectively.

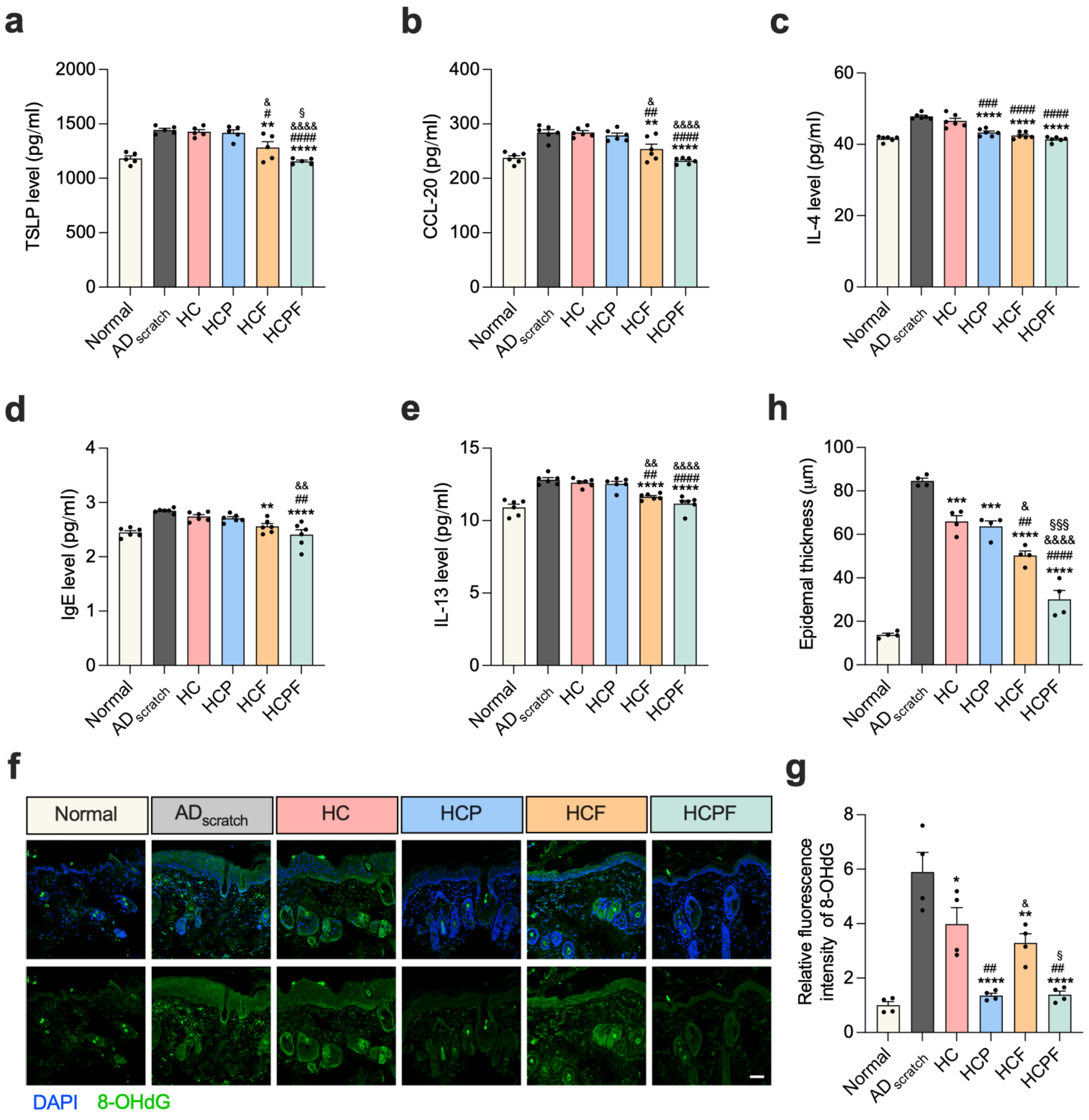

Supplementary Figure 8 | Evaluation of serum factors and oxidative damage in tissues. **a-e**, **Blood** level CCL-20, TSLP, IgE, IL-4 and IL-13 of each group, n = 6 mice for each group. **f**, Fluorescent staining of 8-OHdG (green) and DAPI (blue) in each group. n = 4 mice with similar results. Scale bar, 50 µm. **g**, Fluorescence intensity of 8-OHdG reveals oxidative DNA damage in each group, n = 4 mice. **h**, Epidermal thickness quantified from E-cadherin staining, n = 4 mice. All data are shown as mean ± s.e.m.. *, #, & and § indicates data compared with HC, HCP, and HCF, respectively. *, **, *** and **** indicate $P < 0.05$, $P < 0.01$, $P < 0.001$ and $P < 0.0001$ compared by one-way ANOVA followed by Bonferroni's post hoc test, respectively.

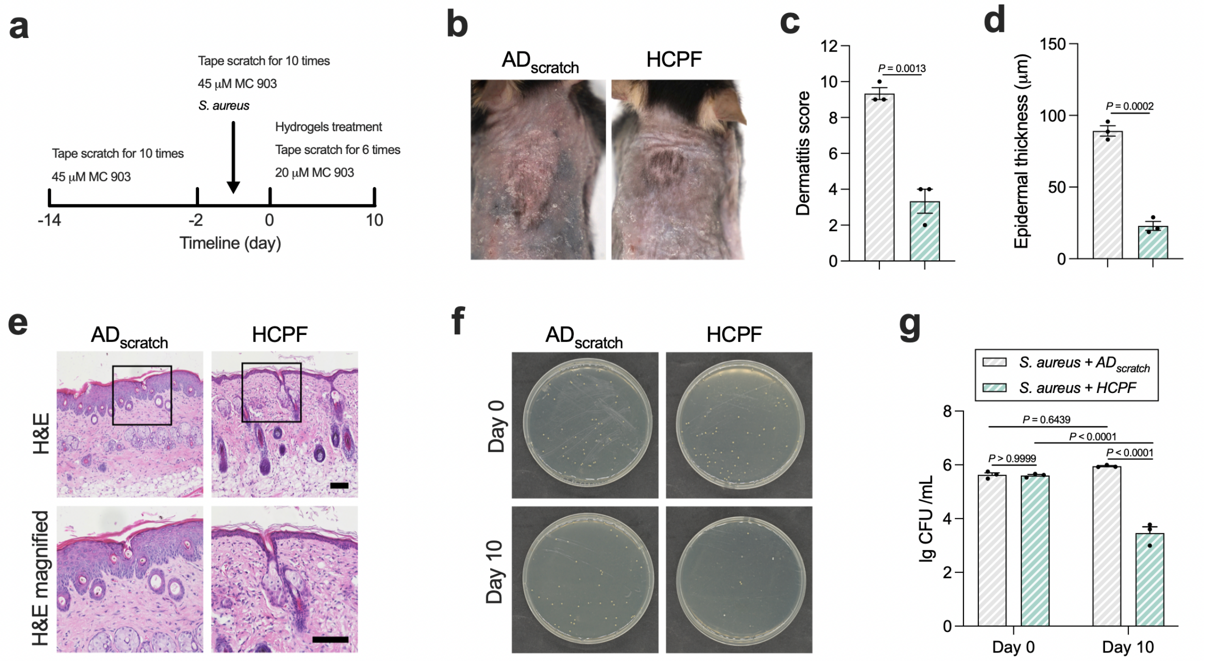

Supplementary Figure 9 | Evaluation of in vivo antibacterial efficiency of HCPF hydrogels.

a, Timeline of *S. aureus* infected animal experiments. **b**, Representative photographs of the dorsal skin of each group. **c**, Dermatitis score of each group assessed from photographs in **b**. **d**, Epidermal thickness quantified from H&E staining. n = 3 mice. In **c** and **d**, n = 3 mice. Data are compared by two-tailed Student's t-test, respectively. **e**, Representative H&E staining of skin section. The black boxed area is enlarged below. Scale bar, 100 μm. n = 3 mice with similar results. **f**, Images of survival *S. aureus* bacteria clones. **g,** lg CFU of *S. aureus* of mice skin calculated from **f**. n = 3 mice. Data are compared by two-way ANOVA followed by Bonferroni's post hoc test, respectively. All data are shown as mean ± s.e.m..
