# Supplementary material for: Hydrogel dressing integrating FAK inhibition and ROS scavenging for mechano-chemical treatment of atopic dermatitis
Source: Nat Commun. 2023 Apr 29;14:2478. doi: 10.1038/s41467-023-38209-x (PMC10148840; doi:10.1038/s41467-023-38209-x)
Supplement: Supplementary file 3 — Reporting Summary [file 41467_2023_38209_MOESM3_ESM.pdf]

nature portfolio

Corresponding author(s): Fneg Xu, Songmei Geng

Last updated by author(s): Apr 11, 2023

Reporting Summary

Nature Portfolio wishes to improve the reproducibility of the work that we publish. This form provides structure for consistency and transparency in reporting. For further information on Nature Portfolio policies, see our Editorial Policies and the Editorial Policy Checklist.

Statistics

For all statistical analyses, confirm that the following items are present in the figure legend, table legend, main text, or Methods section.

n/a	Confirmed	
☐	☒	The exact sample size (*n*) for each experimental group/condition, given as a discrete number and unit of measurement
☐	☒	A statement on whether measurements were taken from distinct samples or whether the same sample was measured repeatedly
☐	☒	The statistical test(s) used AND whether they are one- or two-sided<br>*Only common tests should be described solely by name; describe more complex techniques in the Methods section.*
☒	☐	A description of all covariates tested
☐	☒	A description of any assumptions or corrections, such as tests of normality and adjustment for multiple comparisons
☐	☒	A full description of the statistical parameters including central tendency (e.g. means) or other basic estimates (e.g. regression coefficient) AND variation (e.g. standard deviation) or associated estimates of uncertainty (e.g. confidence intervals)
☐	☒	For null hypothesis testing, the test statistic (e.g. *F*, *t*, *r*) with confidence intervals, effect sizes, degrees of freedom and *P* value noted<br>*Give P values as exact values whenever suitable.*
☒	☐	For Bayesian analysis, information on the choice of priors and Markov chain Monte Carlo settings
☒	☐	For hierarchical and complex designs, identification of the appropriate level for tests and full reporting of outcomes
☒	☐	Estimates of effect sizes (e.g. Cohen's *d*, Pearson's *r*), indicating how they were calculated

Our web collection on statistics for biologists contains articles on many of the points above.

Software and code

Policy information about availability of computer code

Data collection	Confocal images were acquired with the Olympus FV31S-SW (2.3.1.163) software on a Olympus FV3000 microscope.
Data analysis	Data analysis was performed with the GraphPad Prism 9.1, Image J (1.52p) and Image Pro Plus application (version 6.0).

For manuscripts utilizing custom algorithms or software that are central to the research but not yet described in published literature, software must be made available to editors and reviewers. We strongly encourage code deposition in a community repository (e.g. GitHub). See the Nature Portfolio guidelines for submitting code & software for further information.

Data

Policy information about availability of data

All manuscripts must include a data availability statement. This statement should provide the following information, where applicable:

- Accession codes, unique identifiers, or web links for publicly available datasets
- A description of any restrictions on data availability
- For clinical datasets or third party data, please ensure that the statement adheres to our policy

All data in this study are available in the manuscript and the Supplementary materials or from the corresponding author upon reasonable request. Source data are provided with this paper.

Human research participants

Policy information about studies involving human research participants and Sex and Gender in Research.

Reporting on sex and gender	Sex and gender are not considered and important for this study.
Population characteristics	Not relevant for this study.
Recruitment	No recruitment process was done in order to perform presented study. The wax block samples involved were randomly selected by pathologists from patients with atopic dermatitis undergoing biopsy and from patients undergoing cosmetic surgery. All participants received written informed consent.<br>There were no biases in the selection procedure for all samples.
Ethics oversight	The patients signed written informed consent for the use of tissue wax samples before study initiation.

Note that full information on the approval of the study protocol must also be provided in the manuscript.

Field-specific reporting

Please select the one below that is the best fit for your research. If you are not sure, read the appropriate sections before making your selection.

☒ Life sciences ☐ Behavioural & social sciences ☐ Ecological, evolutionary & environmental sciences

For a reference copy of the document with all sections, see nature.com/documents/nr-reporting-summary-flat.pdf

Life sciences study design

All studies must disclose on these points even when the disclosure is negative.

Sample size	No statistical methods were used to predetermine sample sizes. The appropriate sample sizes were determined based on the previously published literature (Nat Biomed Eng 3, 611–620 (2019)) on materials tests and (Nat Med 27, 700–709 (2021)) on animal experiments.<br>For all statistical analyses, three biological replicates was chosen as a self-imposed minimum. The exact replication numbers, sample sizes and statistical methods are described in detail in the text.
Data exclusions	No data were excluded from the analyses.
Replication	All experiments were repeated successfully 3+ times to ensure reproducibility.
Randomization	All samples were randomly allocated into experimental groups.
Blinding	Investigators were blinded to group allocation during data collection and analysis

Reporting for specific materials, systems and methods

We require information from authors about some types of materials, experimental systems and methods used in many studies. Here, indicate whether each material, system or method listed is relevant to your study. If you are not sure if a list item applies to your research, read the appropriate section before selecting a response.

Materials & experimental systems

n/a	Involved in the study	
☐	☒	Antibodies
☐	☒	Eukaryotic cell lines
☒	☐	Palaeontology and archaeology
☐	☒	Animals and other organisms
☒	☐	Clinical data
☒	☐	Dual use research of concern

Methods

n/a	Involved in the study	
☒	☐	ChIP-seq
☒	☐	Flow cytometry
☒	☐	MRI-based neuroimaging

Antibodies

Antibodies used	All antibodies used in this study are listed as follows:<br>1. anti-FAK (1:500, rabbit. Cell Signaling. 71433)

2. anti-Phospho-FAK (1:500, rabbit, Cell Signaling,8556)
3. anti-GAPDH (1:1000, rabbit, Cell Signaling, 5174)
4. anti-E-cadherin (1:1000, rabbit, Cell Signaling, 3195)
5. anti-Filaggrin (1:200, rabbit, Abcam, ab221155)
6. AlexaFluor-488 [H+L] secondary antibodies (1:500, goat anti-rabbit, Cell Signaling, 4412)
7. Anti-8-OHdG (DNA/RNA Damage) antibody (1:100, rabbit, Bioss, bs-1278R)
8. Anti-IgG antibody (1:100, rabbit, Yeasen, 36113ES10)
9. Mouse Interleukin 4 (IL-4) ELISA Kit(mlbio)
10. Mouse Interleukin 13 (IL-13) ELISA Kit(mlbio)
11. Mouse Immunoglobulin E (IgE) ELISA Kit (mlbio)
12. Mouse thymic stromal lymphopoietin (TSLP) ELISA Kit (mlbio)
13. Mouse CCL20 ELISA Kit (mlbio)
14. Human thymic stromal lymphopoietin (TSLP) ELISA Kit (mlbio)
15. Human TNF-α ELISA Kit (mlbio)
16. Human CCL20 ELISA Kit (mlbio)

Validation

All the antibodies were used according to the manufacturer's protocol.
1. anti-FAK (1:500, rabbit. Cell Signaling. 71433)
https://www.cellsignal.cn/products/primary-antibodies/fak-d5o7u-xp-rabbit-mab/71433?site-search-type=Products&N=4294956287&Ntt=fak&fromPage=plp
2. anti-Phospho-FAK (1:500, rabbit, Cell Signaling,8556)
https://www.cellsignal.cn/products/primary-antibodies/phospho-fak-tyr397-d20b1-rabbit-mab/8556?site-search-type=Products&N=4294956287&Ntt=phospho-fak&fromPage=plp
3. anti-GAPDH (1:1000, rabbit, Cell Signaling, 5174)
https://www.cellsignal.cn/products/primary-antibodies/gapdh-d16h11-xp-rabbit-mab/5174?site-search-type=Products&N=4294956287&Ntt=gapdh&fromPage=plp
4. anti-E-cadherin (1:1000, rabbit, Cell Signaling, 3195)
https://www.cellsignal.cn/products/primary-antibodies/e-cadherin-24e10-rabbit-mab/3195?site-search-type=Products&N=4294956287&Ntt=e-cadherin&fromPage=plp
5. anti-Filaggrin (1:200, rabbit, Abcam, ab221155)
https://www.abcam.cn/filaggrin-antibody-epr21892-ab221155.html
6. AlexaFluor-488 [H+L] secondary antibodies (1:500, goat anti-rabbit, Cell Signaling, 4412)
https://www.cellsignal.cn/products/secondary-antibodies/anti-rabbit-igg-h-l-f-ab-2-fragment-alexa-fluor-488-conjugate/4412?site-search-type=Products&N=4294956287&Ntt=4412&fromPage=plp&_requestid=2194665
7. Anti-8-OHdG (DNA/RNA Damage) antibody (1:100, rabbit, Bioss, bs-1278R)
http://www.bioss.com.cn/prolook_03.asp?id=AF08169606000963&pro37=1
8. Anti-IgG antibody (1:100, rabbit, Yeasen, 36113ES10)
https://www.yeasen.com/products/detail/377
9.Mouse Interleukin 4 (IL-4) ELISA Kit(mlbio)
http://www.shjmkit.net/Products-11348651.html
10. Mouse Interleukin 13 (IL-13) ELISA Kit(mlbio)
https://www.mlbio.cn/goods-28504.html
11. Mouse Immunoglobulin E (IgE) ELISA Kit (mlbio)
http://www.shjmkit.net/Products-11332693.html
12. Mouse thymic stromal lymphopoietin (TSLP) ELISA Kit (mlbio)
https://www.mlbio.cn/goods-37281.html
13. Mouse CCL20 ELISA Kit (mlbio)
https://www.mlbio.cn/goods-63497.html
14. Human thymic stromal lymphopoietin (TSLP) ELISA Kit (mlbio)
https://www.mlbio.cn/goods-60509.html
15. Human TNF-α ELISA Kit (mlbio)
https://www.mlbio.cn/goods-77385.html
16. Human CCL20 ELISA Kit (mlbio)
https://www.mlbio.cn/goods-60006.html

Eukaryotic cell lines

Policy information about cell lines and Sex and Gender in Research

Cell line source(s)	HaCaT cells were purchased from ICell Bioscience Inc,Shanghai.
Authentication	Cell lines were authenticated by ICell Bioscience Inc,Shanghai.
Mycoplasma contamination	Cell lines used in these studies tested negative for mycoplasma contamination.
Commonly misidentified lines (See ICLAC register)	No commonly misidentified cell lines were used.

Animals and other research organisms

Policy information about studies involving animals; ARRIVE guidelines recommended for reporting animal research, and Sex and Gender in Research

Laboratory animals	Mouse skin samples were from C57BL/6J male mice of age 6 weeks and were commercially purchased from xi 'an jiaotong university laboratory animal center.<br>Mice were group-housed with the parent mouse on a 12h light-dark cycle, at 22°C with 50% humidity in the air.
Wild animals	Study did not involved wild animals.
Reporting on sex	Sex was not considered in this study. This work presents method for obtaining ELISA data from skin and blood samples and therefore sex is not important criterion.
Field-collected samples	This study did not involve field collected samples.
Ethics oversight	The ethics of animal experiments were approved by the Biomedical Ethics Committee of Xi 'an Jiaotong University Health Science Center (No:2022-1034).

Note that full information on the approval of the study protocol must also be provided in the manuscript.
